# Rapid Shifts in Bacterial Communities and Homogeneity of Symbiodiniaceae in Colonies of *Pocillopora acuta* Transplanted Between Reef and Mangrove Environments

**DOI:** 10.3389/fmicb.2021.756091

**Published:** 2021-10-25

**Authors:** Trent D. Haydon, Justin R. Seymour, Jean-Baptiste Raina, John Edmondson, Nachshon Siboni, Jennifer L. Matthews, Emma F. Camp, David J. Suggett

**Affiliations:** ^1^Climate Change Cluster, University of Technology, Ultimo, NSW, Australia; ^2^Wavelength Reef Cruises, Port Douglas, QLD, Australia

**Keywords:** coral microbiome, 16S rRNA gene, *Pocillopora acuta*, mangrove coral, *Endozoicomonas*, transplant, extreme environment

## Abstract

It has been proposed that an effective approach for predicting whether and how reef-forming corals persist under future climate change is to examine populations thriving in present day extreme environments, such as mangrove lagoons, where water temperatures can exceed those of reef environments by more than 3°C, pH levels are more acidic (pH < 7.9, often below 7.6) and O_2_ concentrations are regularly considered hypoxic (<2 mg/L). Defining the physiological features of these “extreme” corals, as well as their relationships with the, often symbiotic, organisms within their microbiome, could increase our understanding of how corals will persist into the future. To better understand coral-microbe relationships that potentially underpin coral persistence within extreme mangrove environments, we therefore conducted a 9-month reciprocal transplant experiment, whereby specimens of the coral *Pocillopora acuta* were transplanted between adjacent mangrove and reef sites on the northern Great Barrier Reef. Bacterial communities associated with *P. acuta* specimens native to the reef environment were dominated by *Endozoicomonas*, while Symbiodiniaceae communities were dominated by members of the *Cladocopium* genus. In contrast, *P. acuta* colonies native to the mangrove site exhibited highly diverse bacterial communities with no dominating members, and Symbiodiniaceae communities dominated by *Durusdinium.* All corals survived for 9 months after being transplanted from reef-to-mangrove, mangrove-to-reef environments (as well as control within environment transplants), and during this time there were significant changes in the bacterial communities, but not in the Symbiodiniaceae communities or their photo-physiological functioning. In reef-to-mangrove transplanted corals, there were varied, but sometimes rapid shifts in the associated bacterial communities, including a loss of “core” bacterial members after 9 months where coral bacterial communities began to resemble those of the native mangrove corals. Bacterial communities associated with mangrove-to-reef *P. acuta* colonies also changed from their original composition, but remained different to the native reef corals. Our data demonstrates that *P. acuta* associated bacterial communities are strongly influenced by changes in environmental conditions, whereas Symbiodiniaceae associated communities remain highly stable.

## Introduction

Coral reefs worldwide are rapidly deteriorating as a consequence of increasingly frequent and severe marine heatwaves (e.g., Hughes et al., 2018), chronic ocean warming, acidification and deoxygenation (Altieri et al., 2017; Cornwall et al., 2021). Climate change projections indicate that by 2030, most coral reef environments will exceed the current temperature, pH and/or O_2_ thresholds that govern optimal coral reef functioning (Hoegh-Guldberg et al., 2017; Hughes et al., 2018, 2020), threatening reef survival. Understanding whether, and if so how, corals can acclimatise or adapt to these various stressors has therefore become a global research priority in recent years (Howells et al., 2016; Torda et al., 2017; van Oppen and Blackall, 2019; Cropp and Norbury, 2020). One approach has been to study corals already thriving under present-day natural extremes, in reef or reef-adjacent habitats where daily pH, O_2_ and temperature already reach or exceed levels predicted to occur by the end of the century (reviewed by Camp et al., 2018). Such extreme environments include tidal pools (Palumbi et al., 2014; Klepac and Barshis, 2020) and macrotidal reefs (Schoepf et al., 2015, 2020), where corals exhibit exceptional thermal tolerance. Corals in these extreme environments can provide important insights into the future of conspecific reef populations under continued ocean warming.

Extreme systems recently identified as ideal sites for studying coral resilience to complex stressor regimes are reef adjacent mangrove lagoons (Camp et al., 2018), which can be inhabited by species of reef-building corals commonly found on nearby reefs (Macintyre et al., 2000; Rogers, 2009, 2017; Camp et al., 2017, 2019; Lord et al., 2020). Mangroves can reduce thermal stress by shading corals, but these shallow environments also expose corals to hotter, more acidic and deoxygenated conditions (Rogers and Herlan, 2012; Camp et al., 2017; Rogers, 2017; Maggioni et al., 2021). Compared to adjacent reefs, where pH levels fluctuate between 8.1 and 8.2, mangrove lagoons can exhibit consistently low pH (below 7.8), with daily drops to below 7.3 (Camp et al., 2017, 2019). These environments are also on average 2°C warmer than nearby reefs and contain lower dissolved O_2_ concentrations (<1 mg L^–1^) relative to adjacent reefs (Camp et al., 2017, 2020). As a result, corals thriving in mangrove lagoons exhibit very different physiological and metabolic signatures, such as enhanced respiration and reduced photosynthesis (Camp et al., 2017; Ros et al., 2021), which likely facilitate their survival within these relatively hostile environments. However, other underlying factors, such as shifts in the coral microbiome, might also contribute to the persistence of corals in these extreme environments.

Microorganisms are central to healthy coral function (Bourne et al., 2016; Putnam et al., 2017), but their role in coral stress responses has mostly been characterised for the endosymbiotic dinoflagellates (Symbiodiniaceae) (Howells et al., 2012; Cunning et al., 2015a; Lewis et al., 2019; Rosset et al., 2019; Rädecker et al., 2021). Coral hosts closely associated with specific Symbiodiniaceae taxa (Howells et al., 2020; Osman et al., 2020), except when exposed to atypical (“stressful”) conditions (Cunning et al., 2015b; Lewis et al., 2019; Thomas et al., 2019). For example, coral populations persisting across changing environmental gradients experience shifts in dominant Symbiodiniaceae types (Innis et al., 2018; Wall et al., 2020), likely as a host response to retain metabolic compatibility as external resource availability shifts (Suggett et al., 2017). Similarly, corals exhibit ecological associations with diverse and abundant communities of prokaryotes, which play important roles in nutrient cycling (e.g., nitrogen, carbon, sulphur) and protection from pathogens (Ritchie, 2006; Raina et al., 2009; Rädecker et al., 2015; Glasl et al., 2016). The composition of bacterial assemblages associated with corals is rarely static and often displays seasonal (Roder et al., 2015; Sharp et al., 2017) and spatial (McDevitt-Irwin et al., 2017; Ziegler et al., 2019; Osman et al., 2020) heterogeneity governed by changing environmental conditions. There is evidence that these bacterial communities can also facilitate coral acclimation under environmental stress, enhancing coral resilience (Ziegler et al., 2017, 2019; Yu et al., 2020), and recent microbiome manipulation experiments have led to altered levels of stress susceptibility (Fragoso Ados Santos et al., 2015; Damjanovic et al., 2017; Rosado et al., 2019; Doering et al., 2021; Zhang et al., 2021). However, the specific metabolic advantages that beneficial bacteria provide to corals remain to be elucidated.

Recent work in New Caledonia has revealed that conspecific corals inhabiting reef and mangrove habitats harbour different bacterial and Symbiodiniaceae communities (Camp et al., 2020). However, it is unclear whether these contrasting communities are a direct consequence of these different environments, and how plastic these communities might be to environmental variation. Transplantation experiments have been used in reef studies to resolve how corals can adjust to environmental change (and/or populate diverse conditions), demonstrating capacity for corals to acclimatise to depth ranges (Cohen and Dubinsky, 2015; Tamir et al., 2020), temperature regimes (Barshis et al., 2010; Dixon et al., 2018), turbidity gradients (Padilla-Gamiño et al., 2012), and heat stress exposure (Palumbi et al., 2014). Such studies have begun to examine the possible roles that microbial associates play in facilitating acclimation, demonstrating clear shifts in microbial communities when corals are introduced to non-native environments (Ziegler et al., 2019; Chapron et al., 2020; Roitman et al., 2020). For example, when transplanted to a hotter and more thermally variable environment, heat sensitive corals acquired a microbiome similar to that of heat tolerant corals (Ziegler et al., 2017). However, such reshuffling of the microbiome under stress does not appear to be a conserved trait across all coral species because the microbiome of some species remain unchanged when transplanted from unpolluted to polluted environments (Ziegler et al., 2019).

Following recent observations that coral species found on the northern Great Barrier Reef in both mangrove lagoon and adjacent reef sites are inhabited by different communities of Symbiodiniaceae (Low Isles; Camp et al., 2019; Ros et al., 2021), we conducted a 9-month reciprocal transplant experiment, using the common coral species *Pocillopora acuta*, to identify whether: (i) bacterial communities differ between sites, and (ii) bacterial and Symbiodiniaceae community composition changes to resemble that of native corals when moved across the contrasting environments. Given previous observations of rapid adjustments in bacterial assemblages (Sharp et al., 2017; Sweet et al., 2017; Pootakham et al., 2019), we hypothesised that the microbiome of *P. acuta* would shift after 9 months of reciprocal transplantation into non-native sites and would mirror that of native non-transplanted corals. Understanding if the microbiomes of transplanted corals have the capacity to resemble those of native corals is important to decipher some of the possible mechanisms involved of how corals will adapt to changing future conditions.

## Materials and Methods

### Site Description

**FIGURE 1:**
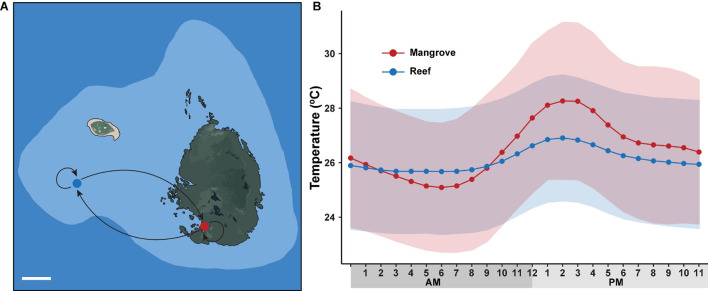
Long-term reciprocal transplantation experiment. **(A)** Map of sampling sites on Low Isles reef crest (blue dot) and adjacent Woody Isles mangrove lagoons (red dot) with different environmental regimes. Five coral colonies were transplanted to each adjacent site (mangrove-reef and reef-mangrove) and five coral colonies remained in their natural environments (mangrove-mangrove and reef-reef). White line (lower left) represents 100 m distance. **(B)** Mean hourly temperature (°C) measured over a 9-month period between May 2018 and February 2019. Data was collected from HOBO sensors deployed on Woody Isles mangrove lagoon (red) and Low Isles reef (blue). Shading represents SD for *n* = 269 per time point.

Two sites, situated ∼500 m apart and located on the northern Great Barrier Reef (GBR), were selected for our reciprocal transplant experiment: a mangrove lagoon (Woody Isles) and an adjacent reef crest-slope (Low Isles reef) (Figure 1). The Woody Isles mangrove lagoon is semi-enclosed by a mangrove forest and undergoes daily tidal flushing, but does not appear to have freshwater inputs from the surrounding catchment. Corals inhabiting this mangrove environment undergo dynamic diurnal changes in temperature (range 7.7°C), pH (1.3), oxygen (7.33 mg l^–1^), and salinity (15.5) (see Camp et al., 2019). In comparison, the Low Isles reef site has considerably more stable physiochemical conditions.

### Experimental Design and Sample Collection

To characterise daily and seasonal temperature shifts, we deployed HOBO Pendant data loggers (Onset, MA, United States) at both sites (Woody Isles mangrove; Low Isles reef), which recorded data for the 9-month duration of the experiment. In addition, spot measurements were periodically taken for temperature, salinity and oxygen using a multi-meter (3430, WTW) and pH using a pH meter (913, Metrohm). Environmental data series (temperature, salinity, O_2_, and pH) were tested using independent samples *t* tests with Welch’s corrections to test for any differences between reef and mangrove environments (GraphPad Prism v.7).

Five colonies of *P. acuta* were selected for transplantation from each habitat. Colonies were sampled from a depth of ca. 0.25–0.5 m (mangrove, *n* = 5) and <1 m (reef crest-slope, *n* = 5) on the lowest astronomical tide to ensure consistency across the two sites. All colonies were subsampled to yield a partial colony fragment, which was then split in half (ca. 10 cm diameter). The two resulting fragments subsequently served as either a native (remaining within site) or non-native (transplanted between sites) specimen, where colonies were either retained within their native mangrove (mangrove-mangrove), or reef (reef-reef), or were moved from the mangrove to the reef (mangrove-reef), or reef to mangrove (reef-mangrove). All split colonies were attached onto one of five aluminium racks with cable ties within each location, such that each rack contained either a replicate mangrove-mangrove or reef-mangrove coral colony within the mangrove lagoon, or replicate reef-reef or mangrove-reef coral colony on the reef (total *n* = 20). Two small fragments (<5 cm each) of *P. acuta* were collected from each of the 20 split colonies at the start of the transplantation (May 2018, t_0_) for characterisation of the Symbiodiniaceae and bacterial communities, and Symbiodiniaceae photo-physiology *via* Pulse Amplitude Modulation (PAM) fluorometry. All fragments were collected using sterile pliers and placed in zip lock bags filled with seawater from the respective location before being returned to the sampling vessel within 30 min for immediate processing. One fragment was used for microbiome characterisation and rinsed with sterile artificial seawater before immediate flash freezing in liquid N_2_, while the second fragment was analysed using PAM fluorometry. Further samples were collected using this approach three days after transplantation (t_3d_) to determine any immediate transplant effects on the coral microbiome or photophysiology. The experiment continued with samples collected 2 months (t_2M_), 3 months (t_3M_), 6 months (t_6M_), and 9 months (t_9M_) after transplantation, which were all immediately processed for microbiome and PAM fluorometry analysis.

### Photophysiological Data

Photophysiology was characterised using a diving PAM set to conduct a fluorescence-light response curve as previously detailed (e.g., Nitschke et al., 2018; Camp et al., 2019), where the actinic light source intensity was verified against a factory-calibrated quantum sensor (Li-COR, United States). Briefly, samples were low light acclimated (ca. 5–10 mol photons m^–2^ s^–1^) for 20 min and then exposed to an increasing light gradient of ca. 0, 180, 210, 360, 450, 670, 1070, 1550, and 1980 μmol photons m^–2^ s^–1^. Minimum (*F*_0_, *F*_0_′, *F*′) and maximum (*F*_*m*_, *F*_*m*_′) fluorescence yields were obtained to calculate the excitation energy dissipation terms [1-C] and [1-Q], which are the photochemical and dynamic non-photochemical coefficients, respectively (see Suggett et al., 2015). To assess the differences of photosynthetic strategies between reef and mangrove corals, data series were confirmed for normality (Shapiro-Wilk) and then tested using independent samples *t* tests (GraphPad Prism v.7).

### Sample Processing and DNA Extractions

Total genomic DNA (gDNA) was extracted using a modified phenol-chloroform protocol. Coral tissue was initially removed from frozen fragments by air-blasting tissue into zip lock bags containing 5 ml of sterile artificial seawater. Coral slurry was then centrifuged at 8,000 *g* for 10 min, the supernatant was discarded, and pellets were frozen at −80°C prior to subsequent analysis. Pellets were later resuspended in 0.5 mL of extraction buffer (0.75 M Sucrose, 40 mM EDTA, 50 mM Tris-base pH 8.3) with the addition of 75 μL of Lysozyme (100 mg/ml stock), incubated at 37°C for 1 h and shaken every 15 min, followed by three freeze/thaw cycles in liquid N_2_ and incubation at 70°C on a heat block. Sodium dodecyl sulphate (SDS; 100 μL of a 25% solution) was added to samples and then incubated at 70°C for 10 min. Samples were cooled to room temperature before adding proteinase K (20 μL of 20 mg/mL stock) and further incubated for 1 h at 37°C, followed by three additional freeze/thaw cycles. Samples were then added to an equal volume of phenol:chloroform:isoamyl alcohol (25:24:1, pH 8), mixed by inversion for ten min at room temperature, followed by centrifugation for 15 min at 13,000 *g*. For each sample, the aqueous phase containing the DNA was transferred into a clean tube and equal volumes of chloroform:isoamyl alcohol (24:1) was added. Samples were mixed by inversion for 10 min and centrifuged for 10 min at 16,000 *g*. The aqueous layer was transferred to a new tube and mixed with sodium acetate (50 μl of 3M solution), then an equal volume of ice-cold molecular-grade isopropanol was added to precipitate the extracted DNA. Tubes were then centrifuged at 20,000 *g* for 30 min at 4°C and the supernatant was discarded. The DNA pellets were washed with 500 μL of molecular grade ethanol (70%) and centrifuged for 10 min 20,000 *g*. Ethanol was removed and samples were air dried for 15 min in the dark, before DNA pellets were resuspended in 30 μL of nuclease free water.

### Host Identification

To confirm the identity of the coral species, extracted DNA was amplified using the Pdam-F (5′-AAGAAGATTCGGGCTCG TTT-′3) and Pdam-R (5′-CGCCTCCTCTACCAAGACAG-′3) primers, which provide species delineation in pocilloporids (Flot and Tillier, 2007). The PCR conditions involved a denaturing cycle at 95°C for 10 min, followed by 25 cycles at 95°C for 30 s, 58.5°C for 30 s, and 72°C for 30 s, and a final extension step at 72°C for 10 min. Amplification efficiency was confirmed using 1% agarose gels with 3 μL of PCR product. Amplicons were then sequenced in both directions using Sanger Sequencing (Australian Genomic Research Facility, NSW, Australia). Sequences were aligned in Geneious V R6 against reference sequences for *Pocillopora* from NCBI. All sequences matched with reference sequences for *P. acuta* (Schmidt-Roach et al., 2014) and confirmed initial classification based on visual identification in the field.

### Prokaryote Community Composition

For characterisation of the bacteria associated with *P. acuta*, the V3-V4 region of the 16S rRNA gene was amplified using the 341F (5′-TCGTCGGCAGCGTCAGATGTGTATAAGAGACAG CCTAYGGGRBGCASCAG-′3) and 805R (5′-GTCTCGTGGG CTCGGAGATGTGTATAAGAGACAGGGACTACNNGGGTAT CTAAT-′3) primers (Klindworth et al., 2013) (underlined segments represent adapter sequences; Illumina, San Diego, CA, United States). PCR reactions consisted of 1 μL of template DNA, 12 μL of Velocity high fidelity master mix (Bioline, United Kingdom) and 1 μL of each primer. The PCR cycling conditions involved an initial denaturation step at 95°C for 10 min, then 25 cycles at 95°C for 30 s, 50°C for 30 s and 72°C for 30 s, followed by a final extension at 72°C for 5 min. Amplicons were sequenced using the Illumina MiSeq platform (2 × 300 bp) at the Ramaciotti Centre for Genomics (University of New South Wales, Sydney, Australia). Raw FASTQ format files obtained from the 16S rRNA gene amplicon sequencing were processed using the Quantitative Insights into Microbial Ecology (QIIME2) pipeline (Bolyen et al., 2019). The DADA2 plugin (version 2019.1.0) was subsequently applied to remove chimeras, denoise and trim paired-end sequences (Callahan et al., 2016). Sequences were examined at the amplicon sequence variants (ASVs) level. ASVs with reads below 0.005% relative abundance and corresponding to chloroplast or mitochondria were removed (Gonzalez et al., 2019) along with any extraction kit contaminants found in sequenced negative controls. Taxonomy was assigned using *classify-sklearn* (Pedregosa et al., 2011) against the SILVA v138 database. Rarefaction curves were produced to determine differences in sequencing depth between samples, with data then rarefied to 5,250 reads per sample. Alpha-diversity indices were produced in QIIME2 (Shannon’s diversity and Chao1 species richness).

To visualise differences in bacterial community composition among locations and treatments, non-metric multidimensional scaling ordinations (nMDS) were carried out using Bray-Curtis dissimilarity matrices. Differences in alpha diversity and community structure (beta diversity) were analysed using permutational multivariate analysis of variance (PERMANOVA) on square-root transformed data in PRIMER-E + PERMANOVA package v1.0.6. One-factorial PERMANOVA was run with 999 permutations to test for differences between sites (mangrove vs reef). Two-factorial PERMANOVAs were run with 999 permutations to test for differences between sites at different timepoints, and differences within sites between timepoints (fixed factors time and site). To identify differentially abundant bacterial taxa across sites and between timepoints, we used MetagenomeSeq (Paulson et al., 2013) at the ASV and family levels. Additionally, to identify the core microbiome of both mangrove and reef environments, we used the panbiom python script (Kahlke, 2018), which detect ASVs present in at least 0.1% relative abundance in more than 80% of samples tested.

### Symbiodiniaceae Community Composition

Symbiodiniaceae community characterisation among *P. acuta* colonies was assessed at t_0_ (*n* = 19) and t_9M_ (*n* = 13) to characterise any changes over time. Specifically, the ITS2 region of the Symbiodiniaceae communities associated with experimental coral colonies was targeted using the ITSintfor2 (5′- TCGTCGGCAGCGTCAGATGTGTATAAGAGACAGGAA TTGCAGAACTCCGTG-′3) and ITS2-reverse (5′-GTCTCGTG GGCTCGGAGATGTGTATAAGAGACAGGGGATCCATATGC TTAAGTTCAGCGGGT-′3) primer pairs (Coleman et al., 1994; LaJeunesse, 2002) (attached Illumina adapters underlined). PCR reactions and sequencing were carried out as described for the 16S rRNA sequencing, except that the annealing temperature used was 55°C. Resulting amplicons were sequenced using the Illumina MiSeq platform (2 × 300 bp) (Australian Genomic Research Facility, Victoria, Australia). Demultiplexed FASTQ files from the Illumina sequencing were analysed using the SymPortal analytical framework (Hume et al., 2019), which predicts ITS2-type profiles from specific sets of defining intragenomic ITS2 sequence variants (DIVs) based on genetically differentiated Symbiodiniaceae taxa. Sequences were submitted directly to the SymPortal pipeline, where they were quality controlled using Mother 1.39.5 (Schloss et al., 2009), BLAST+ suite of executables (Camacho et al., 2009) and minimum entropy decomposition (Eren et al., 2014) to predict Symbiodiniaceae taxa from the ITS2 marker. Differences in Symbiodiniaceae communities were visualised using nMDS and tested using PERMANOVA as described for the prokaryote data analysis above.

## Results

### Environmental Characterisation

Reef and mangrove sites had distinct environmental regimes. Specifically, throughout the 9-month study period, average diel (day-night) temperature variation for the mangroves (28°C) was ∼2°C higher (*p* < 0.001) than for the reef (26°C). Similarly, peak temperature (15:00) was on average 1.5°C higher (*p* < 0.001) in the mangrove (28.2°C) compared to the reef (26.7°) (Figure 1). Over each sampling timepoint (t_0_, t_3D_, t_*t2M*_, t_3M_, t_6M_, and t_9M_), pH, salinity and oxygen were lower in the mangrove (7.74–7.81, 33.9–34.2, 2.5–4.1 mg/L) compared to the reef (8.08–8.11, 35.0–35.2, 6.2–6.8 mg/L) (*p* < 0.0001) (Supplementary Table 1).

### Symbiodiniaceae Associations Are Site-Specific Yet Do Not Change After Transplantation

**FIGURE 2:**
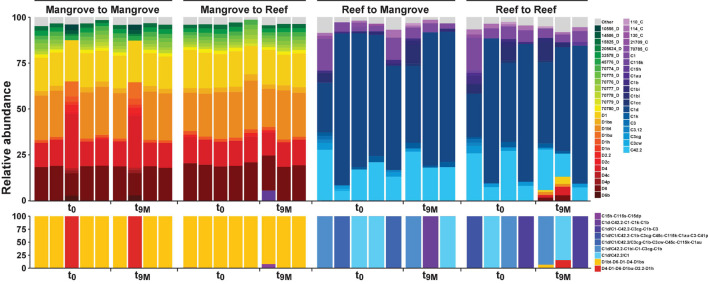
Relative abundance (%) of ITS2 sequence types (upper section) and predicted major ITS2 type profiles (lower section) for *Pocillopora acuta* across both the mangrove lagoons and reef habitats on the Great Barrier Reef over transplantation treatments; mangrove-mangrove (MM), mangrove-reef (MR), reef-mangrove (RM), and reef-reef (RR). t_0_ represents samples taken prior to transplantation and t_9M_ 9 months after transplantation. Only sequence types above 1% relative abundance are shown, anything below 1% relative abundance is represented by top grey bars. Designated names (e.g., D1) represent sequences previously characterised in the literature or that have been previously run through the SymPortal analytical framework (Hume et al., 2019). Other less common sequences designated to a unique database ID and their associated clade/genera (e.g., 10566_D) represent sequences that have not been previously used to characterise ITS2 type profiles. C refers to *Cladocopium* species (clade C) and D refers to *Durusdinium* species (clade D).

**FIGURE 3:**
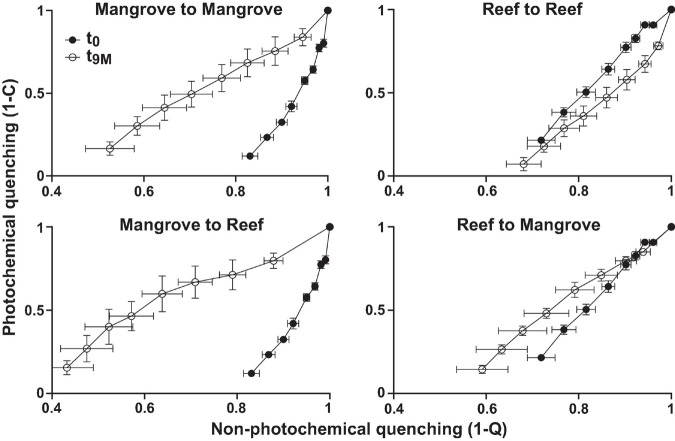
Photophysiology of *Pocillopora acuta* (mean, *n* = 3–5) showing photochemical quenching (1-C) versus non-photochemical quenching (1-Q) from Rapid Light Curves for each transplantation treatment; mangrove-mangrove, mangrove-reef, reef-reef, and reef to mangrove at each sampling timepoint; t_0_ (prior to transplantation) and t_9M_ (after 9 months of transplantation).

Symbiodiniaceae communities associated with *P. acuta* exhibited site-specific ITS2 profiles (*p* < 0.005; Figure 2 and Supplementary Table 2). The Symbiodiniaceae assemblage within mangrove corals was dominated by species from the *Durusdinium* genus (ITS2 type profile D1bt_D6_D1_D4_D1bs and D4_D1_D6_D1bu_D2.2_D1h), while reef corals were dominated by members of the *Cladocopium* genus (ITS2 type profile C1d_C42.2_C1, C1d_C42.2_C1bl_C1_C3cg_C1b and C1d_C1_C42.2_C3cg_C1b_C3), whereby each genus represented >95% of the ITS2 relative abundance at each site. Notably, transplantation to adjacent sites had no effect on coral associated Symbiodiniaceae communities, which remained stable within transplanted corals (Figure 2). After 9 months (t_9M_), ITS2 profiles of the transplants (either within or between sites) were indistinguishable from those at the beginning of the experiment (t_0_) (*p* > 0.05; Supplementary Table 3). Photo-physiological assessments revealed strong seasonal signatures in the dissipation of excitation energy for the mangrove corals, which were retained independently of transplanted location (i.e., mangrove-mangrove and mangrove-reef) (Figure 3). Specifically, much greater non-photochemical quenching [lower values of (1-Q)] occurred at t_9M_ when water was the warmest, compared to t_0_ (*p* < 0.001). In contrast, reef populations exhibited no significant change in quenching dynamics through time, a pattern that was conserved whether colonies were located in native (reef-reef) or non-native (reef-mangrove) habitats (Figure 3). Thus, Symbiodiniaceae communities also retained their initial physiological signatures regardless of location over the 9-month period following transplantation.

### Sequencing Overview

In total, 113 samples of *P. acuta* (t_0_; *n* = 20, t_3D_; *n* = 19, t_2M_; *n* = 20, t_3M_; *n* = 18, t_6M_; *n* = 19, t_9M_; *n* = 14) and 3 negative controls were characterised using 16S rRNA amplicon sequencing, which yielded 10,745,579 sequences (Supplementary Table 4). Following quality control and removal of unwanted sequences, we retained 3,241,809 sequences (mean ± SE, 28,688 ± 7951 per sample). After Subsampling to 5250 sequences per sample, there were 110 samples retained with a total of 13,380 ASVs in the dataset.

### Coral-Associated Bacterial Communities Are Site-Specific

**FIGURE 4:**
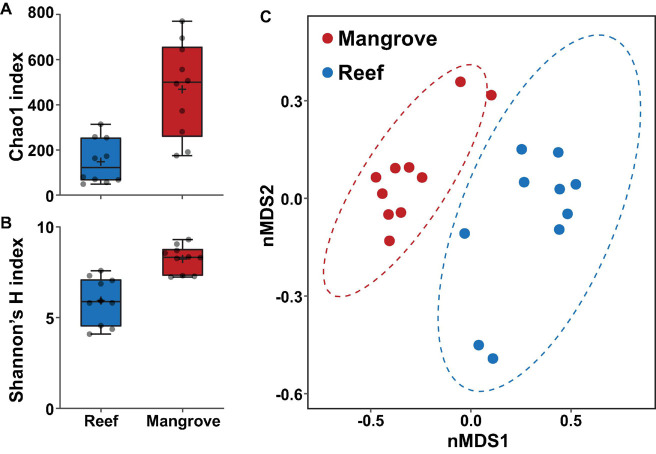
Bacterial diversity of ASVs associated with *Pocillopora acuta* in the mangrove lagoons and the reef environments prior to transplantation of corals (t_0_), based on **(A)** Chao1 and **(B)** Shannon’s diversity index. Box plots represent 25th–75th percentile range, lines show medians, error bars represent IQR and + represent the mean, *n* = 10 coral colonies. **(C)** Bacterial community structure at the ASV level within the coral *Pocillopora acuta* between mangrove lagoon (*n* = 10) and reef environments (*n* = 10) at t_0_. Plot is based on non-metric multidimensional scaling (nMDS) of Bray-Curtis distances with corals prior to transplantation. Ellipses denote 95% confidence intervals. 2D stress: 0.10.

Bacterial alpha diversity analysis revealed statistically significant patterns between environments (PERMANOVA, *p* < 0.001; Supplementary Table 5). Specifically, bacterial community richness at t_0_ was significantly higher in the mangrove corals relative to corals located on the reef (ASV richness of 469 ± 66 SE and 149 ± 31, respectively; Figure 4A), and more diverse than in the reef (Shannon diversity of 8.2 ± 0.23 and 5.9 ± 0.40, respectively; Figure 4B).

**FIGURE 5:**
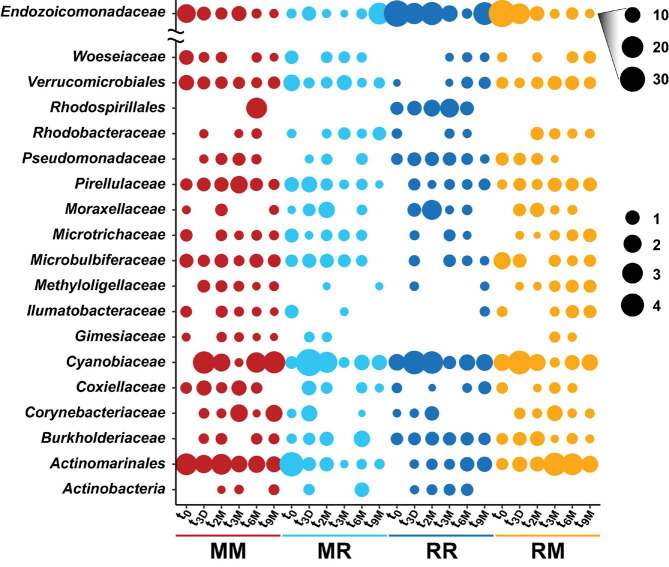
Bubble plot of the highest abundant bacterial families associated with the coral *Pocillopora acuta* across different collection timepoints (t_0_; 0 days, t_3D_; 3 days, t_2M_; 2 months, t_3M_; 3 months, t_6M_; 6 months, t_9M_; 9 months) and transplant treatments (MM; mangrove-mangrove, MR; mangrove-reef, RR; reef-reef and RM; reef-mangrove). Size of the bubble represents the relative abundance. Note: Due to their abundance, bubble sizes of Endozoicomonadaceae were scaled separately.

**FIGURE 6:**
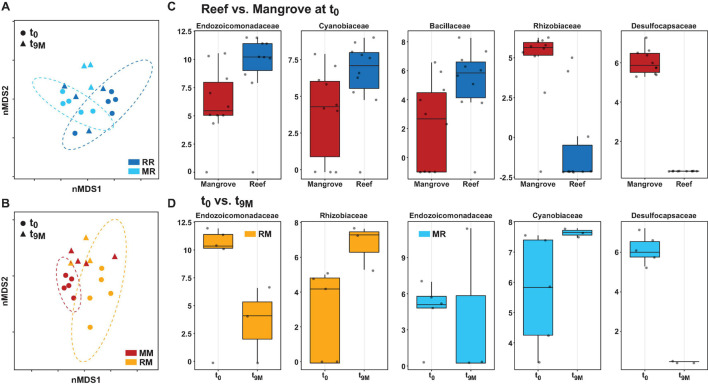
Bacterial community structure at the amplicon sequence variant (ASV) level and differential abundance of bacterial families associated with the coral *Pocillopora acuta.* Non-metric multidimensional scaling (nMDS) of Bray-Curtis distances between timepoints (t_0_ and t_9M_) and treatments; **(A)** reef-reef (RR) and mangrove-reef (MR) (2D stress: 0.18), **(B)** mangrove-mangrove (MM) and reef-mangrove (RM) (2D stress: 0.13). MetagenomeSeq analysis of significantly differentially abundant bacterial families (*p* < 0.05 following FDR corrections) between **(C)** native reef and mangrove corals (t_0_), **(D)** timepoints in mangrove-reef (MR) corals (t_0_ vs t_9M_) and reef-mangrove (RM) corals (t_0_ vs t_9M_). Bacteria family names are provided above each box, box plots represent log transformed count.

The composition of the bacterial communities was also site-specific (PERMANOVA, *P* < 0.001; Figure 5, Supplementary Figure 1, and Supplementary Table 6). This was further confirmed visually by ordination analysis (Figure 4C). These inter-site differences at t_0_ were driven by 79 differentially abundant ASVs, however, each of these ASVs was generally rare (<1% relative abundance). Therefore, we further analysed these differences at the family level using metagenomeSeq. Overall, we identified 60 differentially abundant bacterial families between the two sites (Supplementary Table 7), but only five of these represented at least 2% relative abundance in samples of either habitat (mangrove or reef). Specifically, Endozoicomonadaceae (33% ± 8.7), Cyanobiaceae (6% ± 1.7), and Bacillaceae (2.8% ± 0.78) were relatively more abundant in the reef corals at t_0_, whereas Rhizobiaceae (2.6% ± 0.2) and Desulfocapsaceae (2.5% ± 0.3) were relatively more abundant in the mangrove corals at t_0_ (Figure 6C).

### Coral Core Microbiome

At t_0,_ a core microbiome was not conserved across *P. acuta* colonies in the mangrove environment. In contrast, 5 ASVs were identified as core members of the microbiomes in reef colonies. These 5 ASVs all belonged to the genus *Endozoicomonas*, on average they accounted for 26.3% of the microbiome in reef colonies and were all present in 9 out of the 10 replicates at t_0_.

### Bacterial Communities Rapidly Shift When Corals Are Transplanted

**FIGURE 7:**
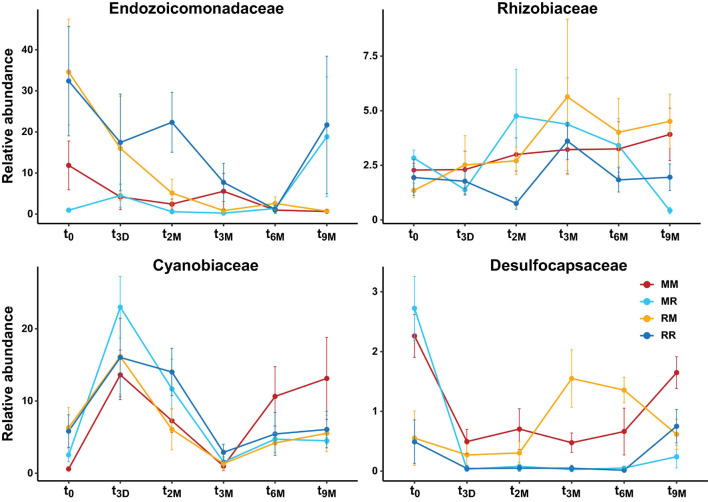
Changes in relative abundance of dominant bacterial families over time (t_0_; 0 days, t_3D_; 3 days, t_2M_; 2 months, t_3M_; 3 months, t_6M_; 6 months, t_9M_; 9 months) and between transplant treatments (MM; mangrove-mangrove, MR; mangrove-reef, RM; reef-mangrove, and reef-reef; RR). Error bars represent standard error.

Transplantation of *P. acuta* colonies from the reef to the mangrove lagoon (reef-mangrove) resulted in no significant changes in alpha diversity over time (PERMANOVA, *p* > 0.05; Supplementary Figure 2, Supplementary Table 8). However, comparisons of beta diversity revealed a significant interactive effect between sites and timepoints (PERMANOVA, *p* < 0.005; Supplementary Table 9). Composition of bacterial communities in reef-mangrove corals shifted after 3 months of transplantation (t_3M_), where these samples as well as those from subsequent time points, became highly dissimilar compared to t_0_ samples (PERMANOVA, *p* < 0.05; Figure 5 and Supplementary Table 9). In addition, communities between t_0_ and t_9M_ were clearly partitioned (nMDS; Figure 6B). These changes in bacterial communities from t_0_ to t_9M_ were primarily driven by statistically significant shifts in the relative abundance of 17 bacterial families (Supplementary Table 10). Notably, the proportion of Endozoicomonadaceae dramatically decreased from an average of 35 to 0.7%, while the relative abundance of members of the Rhizobiaceae significantly increased over time (1.3–4.5%) (Figures 6D, 7).

Coral-associated bacterial communities in corals transplanted from mangrove to reef (mangrove-reef) were significantly altered after only three days (t_3D_) of transplantation (PERMANOVA, *p* < 0.05; Supplementary Table 9). Overall ASV diversity (Shannon diversity) decreased over time (t_0_; 7.9 and t_9M_; 5.1, *p* < 0.05; Supplementary Figure 2 and Supplementary Table 8), but no significant changes were observed in bacterial richness. Changes in bacterial community structure were observed between t_0_ and t_9M_ (nMDS; Figure 6A) along with overall changes in community composition (PERMANOVA, *p* < 0.05, Figure 5 and Supplementary Table 9). Among mangrove-reef colonies, nine bacterial families were differentially abundant over time (t_0_–t_9M_) (Supplementary Table 11), specifically significant increases in the relative abundance of Endozoicomonadaceae (0.96–19%) and Cyanobiaceae (2.5–4.5%), and a decrease in Desulfocapsaceae (2.7–0.24%) occurred (Figures 6D, 7). Each individual mangrove-reef replicate became highly dominated by one or two different bacterial families following 9 months of transplantation to the reef. For example, one of the replicates was characterised by a high relative abundance of Rhodobacteraceae (79%), whereas another was dominated by Endozoicomonaceae (56%) (Supplementary Figure 1). This pattern contrasts with the lack of many highly relatively abundant bacterial families (>5%) among mangrove-reef samples prior to transplantation (t_0_).

### Coral Microbiomes of Transplanted Corals Resemble Those of Native Corals

Transplantation of coral colonies between habitats indicated the rate with which coral microbial communities adjusted to their new sites (environmental regime). The bacterial assemblages associated with the corals transplanted from the reef to mangrove (reef-mangrove) became indistinguishable to those of the native mangrove corals (mangrove-mangrove) by t_9M_ (PERMANOVA, *p* > 0.05; nMDS; Figure 6B). In contrast, the bacterial communities associated with corals that remained in the reef environment (reef-reef) did not change significantly over the course of the experiment. Bacterial communities of the mangrove-reef corals changed over time but showed little resemblance to the bacterial communities of the native reef corals (reef-reef) by t_9M_ (nMDS; Figure 6A). Additionally, the bacterial communities in the native mangrove corals (mangrove-mangrove) shifted between t_0_ and t_9M_ (*p* < 0.05), which was characterised partly by the loss of *Endozoicomonas* ASVs (Figure 7). In fact, bacterial communities within the mangrove–mangrove native colonies were highly dynamic and shifted significantly at every timepoint measured (PERMANOVA, *p* < 0.05; Figure 5 and Supplementary Table 9). The major distinction between bacterial communities from t_0_ to t_9M_ for the native mangrove corals was the loss of highly abundant bacterial families, which contrasted with the corals translocated from mangrove to the reef (Figure 5).

## Discussion

Coral microbiomes are sensitive to environmental stressors and can dramatically differ across colonies of the same coral species inhabiting dissimilar environments (McDevitt-Irwin et al., 2017). Within this context, there is evidence that corals tolerant of the often hot, acidic and deoxygenated conditions within “extreme” mangrove lagoons harbour different microbial communities relative to colonies of the same species on nearby reefs (Camp et al., 2020). However, whether corals from mangrove environments retain such microbial characteristics when transplanted to a more stable reef environment is currently unknown. Here, we performed the first reciprocal transplant experiment of corals between a mangrove lagoon and adjacent reef, to identify the influence of environmental extremes on the structure of the coral microbiome. Our results show that a rapid shift in the bacterial communities occurred after translocation, whereas the Symbiodiniaceae communities – and associated physiological performance – remained remarkably stable. Bacterial communities associated with corals translocated from the reef environment into mangrove habitats became statistically indistinguishable from native mangrove corals. In contrast, bacterial communities of corals moved from the mangrove to reef environment did not begin to resemble the native reef corals, even 9 months after transplantation. Our experiment demonstrates that the bacterial communities of *P. acuta* strongly respond to changing environmental conditions, but the pressure exerted on the bacterial community is greater in mangrove environments.

### Reef Versus Mangrove Environmental Conditions Influence Coral-Microbial Associations

Symbiodiniaceae taxa profoundly influence the physiology and stress resilience of their coral hosts (Oliver and Palumbi, 2011; Howells et al., 2012; Suggett et al., 2017). We found distinct communities of Symbiodiniaceae associated with *P. acuta* between the reef and mangrove environments, which is in line with previous observations at this field site (Ros et al., 2021). *P. acuta* colonies on the reef were largely dominated by the genus *Cladocopium*, whereas the mangrove colonies were dominated by the genus *Durusdinium.* Whilst *Durusdinium* is often observed in corals with enhanced stress tolerance, in particular to increased temperature (Berkelmans and Van Oppen, 2006; Lajeunesse et al., 2014; Hoadley et al., 2019), this genus is also often abundant with corals inhabiting turbid reefs and shallow high-light environments (Hoadley et al., 2019; Wall et al., 2020). However, it appears that other coral species in mangrove lagoons are not predominately associated with *Durusdinium*, but instead with ITS2 types corresponding to *Cladocopium* [e.g., *Porites lutea* (Woody Isles; Camp et al., 2019), *Acropora muricata* and *Acropora pulchra* (New Caledonia; Camp et al., 2020)]. Our contrasting findings for *P. acuta* suggest that Symbiodiniaceae-coral associations in multi-stress mangrove environments is species specific.

Symbiodiniaceae communities remained stable over time in both the reef and mangrove corals, which is consistent with previous studies in reef environments (Klepac et al., 2015; Cai et al., 2018; Epstein et al., 2019). However, it is perhaps surprising that after 9 months of reciprocal-transplantation, the seasonally dependent photo-physiological signatures of these Symbiodiniaceae communities remained unchanged from their native state. Corals have been known to shift their dominant Symbiodiniaceae compositions from *Cladocopium* to *Durusdinium* following changes in environmental conditions (Jones et al., 2008; Cunning et al., 2018). However, the conserved association observed after transplantation in this study reveals a high capacity to tolerate the new environmental regimes over 9 months. Transplantation began during the beginning of winter (t_0_, May 2018) when the various environmental conditions between the two sites were more similar than towards the end of the experiment in summer (t_6__–__9M_, December 2018–February 2019) (Supplementary Table 1), suggesting the change in conditions were slow enough for acclimatisation of the inherent Symbiodiniaceae-host association.

Similar to the Symbiodiniaceae communities, coral-associated bacterial communities differed between *P. acuta* colonies inhabiting mangrove and reef environments. Such difference among sites is consistent with previous studies that have identified highly divergent bacterial communities in *P. acuta* over small spatial scales (Wainwright et al., 2019; Deignan and McDougald, 2021). However, despite the dynamic nature of *P. acuta* microbiomes over small geographical scales, *P. acuta* microbiomes appear to remain relatively stable under thermal stress (Epstein et al., 2019). One notable feature among mangrove *P. acuta* colonies was the exceptionally high diversity of bacteria observed at both the ASV and genus levels. Compared to other reported values (Shannon’s index 1.5–3.5) in previous *P. acuta* studies (Epstein et al., 2019; Wainwright et al., 2019), alpha diversity values in our study were more than twofold higher. High bacterial diversity (Simpson’s index 75) has also been observed in some mangrove corals in New Caledonia, but these patterns are not consistent across all mangrove coral species, with some exhibiting much lower values (Simpson’s index 1–4) (Camp et al., 2020). Additionally, the *P. acuta* colonies within the mangroves lacked a core microbiome and only a few members of the bacterial assemblage exceeded 5% average relative abundance. Compared to the oligotrophic and often stable conditions of tropical reefs, mangrove environments are highly heterogeneous, with dynamic physiochemical shifts, and impacts from higher nutrient loads and organic content (Huxham et al., 2010; Camp et al., 2017; De Valck and Rolfe, 2018). These more heterogenous conditions likely favour a more diverse microbial community that would conceivably harbour greater responsiveness to changing environmental conditions than a microbiome dominated by only a few species (Roder et al., 2015).

In contrast to the lack of a core microbiome among *P. acuta* colonies inhabiting the mangrove environment, five ASVs belonging to the *Endozoicomonas* genus were identified as members of a core microbiome in the reef colonies. *Endozoicomonas* was also the most relatively abundant bacterial genus in reef corals, a common observation in *P. acuta* (Epstein et al., 2019; Damjanovic et al., 2020a,b) and many other coral microbiome studies (Bayer et al., 2013; Neave et al., 2017; Pogoreutz et al., 2018; Glasl et al., 2019; Ziegler et al., 2019; Haydon et al., 2021). While *Endozoicomonas* was also present in mangrove corals, there was a large difference in the total relative abundance of this genus between the two environments (reef 40%, mangrove 7%). This is consistent with previous reports from corals in mangroves and adjacent reefs in New Caledonia (Camp et al., 2020).

### Transplanted Corals Undergo Rapid Shifts in Bacterial Community Composition and Loss of Core Communities Over Time

After 9 months of transplantation (t_9M_), coral associated bacterial communities in the native transplants (reef-reef) underwent very little change in composition, suggesting there had been little to no effect of transplantation on the bacterial communities associated with these corals. In contrast, the bacterial communities associated with the mangrove-mangrove corals differed significantly over all six timepoints sampled, highlighting a dynamic relationship between bacterial communities and the environment.

The bacteria communities associated with both groups of cross-habitat transplanted colonies (reef-mangrove, mangrove-reef) underwent strong shifts over the 9-month duration of the experiment. This pattern is in contrast to previous studies, that have shown that the bacterial communities associated with *Pocillopora* corals (*P. acuta* or the closely related *Pocillopora verrucosa*) remain relatively stable even when subjected to dramatic changes in environmental conditions, including temperature or pollution (Sawall et al., 2015; Pogoreutz et al., 2018; Epstein et al., 2019; Ziegler et al., 2019). Changes in bacterial communities were apparent after only 3 days in the mangrove-reef corals. Such rapid changes in bacterial community structure indicates plasticity among mangrove corals but also outlines the presence of strong environmental pressure on the microbiome.

Extreme fluctuations over diel and tidal cycles at Woody Isles (Camp et al., 2019) and other mangrove lagoon systems (Camp et al., 2017), as well as the enriched nutrient conditions present in mangrove environments compared to adjacent reefs (De Valck and Rolfe, 2018), are likely underlying factors for the profound changes observed in reef-mangrove *P. acuta* associated bacterial communities. Coral microbiomes can be altered by shifting environmental conditions such as nutrient availability (Zaneveld et al., 2016; Ziegler et al., 2019), temperature (Wang et al., 2018; Gardner et al., 2019), pH (Webster et al., 2016; Grottoli et al., 2018), and salinity (Röthig et al., 2016). In addition, internal physiochemical conditions of corals with thin tissue (e.g., *Pocillopora*) are more influenced by the external environment compared to those with thicker tissue (Putnam et al., 2017). Therefore, given the short generation times of bacteria and their ability to respond to changes in environmental conditions and physiochemical gradients at a rapid rate, we propose that the inherently high environmental variations in mangrove lagoons promotes changes in the bacterial composition of transplanted corals as well as in the native mangrove colonies.

Changes in bacterial communities in reef-mangrove corals after transplantation were largely characterised by a rapid decrease in relative abundance, and potential loss, of *Endozoicomonas*. These patterns are notable, given the prominence, and potentially important function, of members of this genus in coral microbiomes (Neave et al., 2016, 2017). Our findings are consistent to other studies that have observed significant reductions in the relative abundance of *Endozoicomonas* under changing environmental conditions that promote coral stress (Bourne et al., 2008; Roder et al., 2015; Ziegler et al., 2016; Gignoux-Wolfsohn et al., 2017). While a range of changing environmental conditions may be responsible for the decreased abundance of this bacteria in reef-mangrove colonies, *Endozoicomonas* is thought to be particularly sensitive to changes in pH (Morrow et al., 2015). One study found that coral-associated *Endozoicomonas* were significantly reduced at low pH (7.9), suggesting that a decrease in pH could even result in the loss of *Endozoicomonas* (Webster et al., 2016). Following 3 months of transplantation (t_3M_), when we observed a significant shift in the bacterial communities of Reef-mangrove colonies from their original state, the pH in the mangrove environment (pH 7.8) was 0.3 units lower than in the reef environment (pH 8.1). The acidic conditions of the mangrove environment are likely responsible for the loss of other coral-associated bacteria among reef corals.

### Corals Transplanted From the Reef to the Mangrove Develop Bacterial Communities That Resemble Native Mangrove Corals

In line with recent studies in thermally variable habitats (in *Acropora hyacinthus*) and polluted sites (in *Acropora hemprichii*) (Ziegler et al., 2017, 2019), transplanted coral-associated bacteria communities (reef-mangrove) became similar to those of the native transplanted corals (mangrove-mangrove) by the end of the experiment. Besides the observed shifts in the relative abundance of bacterial communities in reef-mangrove colonies there was also an acquisition of many new low abundant bacteria (<1%) that were not present prior to transplantation. An increase in bacterial diversity is thought to be indicative of a holobiont stress response (Thurber et al., 2009; Zaneveld et al., 2017), whereby opportunists shift species dominance away from symbiotic bacterial members. However, considering the high bacterial diversity of the native mangrove corals (mangrove-mangrove), this is perhaps more suggestive of local environmental conditions selecting for a more metabolically diverse community of bacteria (Kelly et al., 2014), which may be beneficial to the host under variable environmental conditions. In contrast to the patterns seen in the reef-mangrove transplants, the bacterial communities of mangrove-reef transplanted corals did not resemble the native reef-reef colonies by the end of the experiment. We did observe a significant decrease in bacterial diversity in mangrove-reef corals between the beginning of the experiment and 9 months later, characterised with a divergence from their original state, but no resemblance to the native reef corals. However, each individual replicate was dominated by different bacteria, suggesting an acclimation response and stabilised microbial community function in each coral colony. Thus, the relatively stable conditions on the reef may have played an important role in selecting for the increased abundance of specific bacteria.

## Conclusion

Here we examined the microbial (Symbiodiniaceae and bacterial) communities associated with *P. acuta* in mangrove lagoon and adjacent reef, and characterised the effect of the surrounding environment on the structure of the microbiome by transplanting corals between the sites. We show that corals living in extreme mangrove environments support highly diverse and transient bacterial communities, which could be beneficial and allow them to cope under environmental variability. In comparison, reef corals maintained more consistent bacterial communities with highly dominant members. Transplantation of corals induced major shifts in bacterial composition, but had little to no effect on the Symbiodiniaceae communities. Our results reveal that coral-associated Symbiodiniaceae communities are initially shaped by divergent environmental conditions, but appeared unaffected by longer term (9 month) changes to these conditions. Bacterial communities changed rapidly following transplantation, suggesting strong selective pressure from the environment. The dynamic nature of the bacterial communities in resident mangrove corals reflects that of the microbiomes of corals persisting in other extreme environments – such as *A. hemprichii*, *A. hyacinthus*, and *P. verrucosa* – which have exhibited acclimation and adaptation potential (Ziegler et al., 2017, 2019). This research demonstrated the dynamic nature of *P. acuta* bacterial communities and highlights the strong influence of changing environmental conditions on their community structure.

## Data Availability Statement

Raw fastq read files were deposited in the NCBI Sequence Read Archive (SRA), accession number: PRJNA764039.

## Author Contributions

DS and EC designed the experiments. EC, TH, DS, and JE collected and processed the samples. TH performed all laboratory work and data analysis. TH and J-BR produced the figures. TH, JS, J-BR, DS, and EC wrote the manuscript. All authors edited the manuscript.

## Conflict of Interest

The authors declare that the research was conducted in the absence of any commercial or financial relationships that could be construed as a potential conflict of interest.

## Publisher’s Note

All claims expressed in this article are solely those of the authors and do not necessarily represent those of their affiliated organizations, or those of the publisher, the editors and the reviewers. Any product that may be evaluated in this article, or claim that may be made by its manufacturer, is not guaranteed or endorsed by the publisher.
