# Supplementary material for: Rapid Shifts in Bacterial Communities and Homogeneity of Symbiodiniaceae in Colonies of Pocillopora acuta Transplanted Between Reef and Mangrove Environments
Source: Front Microbiol. 2021 Oct 25;12:756091. doi: 10.3389/fmicb.2021.756091 (PMC8575411; doi:10.3389/fmicb.2021.756091)
Supplement: Supplementary file 1 [file Data_Sheet_1.DOCX]

Supplementary Material

**Rapid shifts in bacterial communities and homogeneity of Symbiodiniaceae in colonies of *Pocillopora acuta* transplanted between reef and mangrove environments**

Trent D Haydon^1^*, Justin R Seymour^1^, Jean-Baptiste Raina^1^, John Edmondson^2^, Nachshon Siboni^1^, Jennifer Matthews^1^, Emma F Camp^1^, David J Suggett^1^

**Supplementary Figures and Tables**

**Supplementary Table S1.** Environmental parameters in the mangrove and reef environments at each time point ( t_0_, t_3D_, t_2M_, t_3M_, t_6M_, t_9M_) during the nine month transplant experiment. N=3 for pH, salinity, oxygen and temperature measurements, SE = standard error of the mean.

| **Mangrove** | | | | | | | | |
| --- | --- | --- | --- | --- | --- | --- | --- | --- |
|  | **pH** | | **Salinity** | | **O2 (mg/L)** | | **Temp (°C)** | |
|  | **Average** | **SE** | **Average** | **SE** | **Average** | **SE** | **Average** | **SE** |
| **t_0_** | 7.759 | 0.005 | 34.167 | 0.033 | 3.180 | 0.020 | 24.4 | 0.066 |
| **t_3D_** | 7.744 | 0.007 | 34.200 | 0.000 | 3.303 | 0.124 | 24.4 | 0.01 |
| **t_2M_** | 7.781 | 0.014 | 33.933 | 0.033 | 3.043 | 0.003 | 24.0 | 0.066 |
| **t_3M_** | 7.807 | 0.004 | 34.000 | 0.000 | 4.106 | 0.012 | 23.0 | 0.033 |
| **t_6M_** | 7.625 | 0.012 | 34.167 | 0.033 | 2.516 | 0.013 | 29.5 | 0.066 |
| **t_9M_** | 7.717 | 0.007 | 33.900 | 0.000 | 3.393 | 0.308 | 29.8 | 0.033 |
| **Reef** | | | | | | | | |
|  | **pH** | | **Salinity** | | **O2 (mg/L)** | | **Temp (°C)** | |
|  | **Average** | **SE** | **Average** | **SE** | **Average** | **SE** | **Average** | **SE** |
| **t_0_** | 8.064 | 0.012 | 35.133 | 0.067 | 6.37 | 0.040 | 24.1 | 0.00 |
| **t_3D_** | 8.100 | 0.005 | 35.100 | 0.000 | 6.18 | 0.055 | 24.1 | 0.06 |
| **t_2M_** | 8.079 | 0.001 | 35.033 | 0.033 | 6.80 | 0.046 | 23.7 | 0.03 |
| **t_3M_** | 8.111 | 0.005 | 35.167 | 0.033 | 6.52 | 0.03 | 22.8 | 0.03 |
| **t_6M_** | 8.084 | 0.006 | 35.033 | 0.033 | 6.62 | 0.035 | 28.3 | 0.00 |
| **t_9M_** | 8.095 | 0.005 | 35.167 | 0.067 | 6.44 | 0.037 | 28.9 | 0.03 |

**Supplementary Table S2.** PERMANOVA analysis of Symbiodiniaceae communities associated with *Pocillopora acuta* between mangrove (n=10) and reef environments (n=9) prior to transplantation (t_0_). df = degrees of freedom; SS = sum of squares; MS = mean sum of squares; Pseudo-F = F-value by permutation, *p* = *p*-value

| **Source** | **df** | **SS** | **MS** | **Pseudo-F** | **R^2^** | **P (perm)** |
| --- | --- | --- | --- | --- | --- | --- |
| **Sites** | 1 | 37985 | 37985 | 39.861 | .996 | .001 |
| **Residuals** | 18 | 171.53 |  |  |  |  |

**Supplementary Table S3.** Two-factorial PERMANOVA analysis of Symbiodiniaceae communities associated with *Pocillopora acuta* between transplantation time points (t_0_ and t_9M_) of treatments; mangrove-mangrove (MM), mangrove-reef (MR), reef-reef (RR) and reef-mangrove (RM). df = degrees of freedom; SS = sum of squares; MS = mean sum of squares; Pseudo-F = F-value by permutation, *p* = *p*-value.

| **Main PERMANOVA Test** | | | | | | | **Pairwise (t_0_-t_9M_)** | | |
| --- | --- | --- | --- | --- | --- | --- | --- | --- | --- |
| **Source** | **df** | **SS** | **MS** | **Pseudo-F** | **R^2^** | ***p* (perm)** | **Groups** | ***t*** | ***p* (perm)** |
| **Timepoint** | 1 | 354.38 | 354.38 | 0.47304 | .005 | .712 | MM | 0.12869 | 0.948 |
| **Site** | 3 | 64607 | 21536 | 28.747 | .982 | 0.001 | MR | 0.7637 | 0.636 |
| **Timepoint x Site** | 3 | 652.26 | 217.42 | 0.29022 | .010 | 0.987 | RR | 0.64504 | 0.691 |
| **Residuals** | 25 | 187.29 |  |  |  |  | RM | 0.59985 | 0.677 |

**Supplementary Table S4.** Summary of sequence counts from 16S rRNA Illumina sequencing in bacterial communities of *Pocillopora acuta* samples among treatments; mangrove-mangrove (MM), mangrove-reef (MR), reef-reef (RR) and reef-mangrove (RM).

| **Sample replicates** | **Number of raw sequences** | **Treatment** | **Site of origin** |
| --- | --- | --- | --- |
| \| t0-1 \| \| --- \| \| t0-2 \| \| t0-3 \| \| t0-4 \| \| t0-5 \| \| t0-6 \| \| t0-7 \| \| t0-8 \| \| t0-9 \| \| t0-10 \| \| t0-11 \| \| t0-12 \| \| t0-13 \| \| t0-14 \| \| t0-15 \| \| t0-16 \| \| t0-17 \| \| t0-18 \| \| t0-19 \| \| t0-20 \| \| t3D-1 \| \| t3D-2 \| \| t3D-3 \| \| t3D-4 \| \| t3D-5 \| \| t3D-6 \| \| t3D-7 \| \| t3D-9 \| \| t3D-10 \| \| t3D-11 \| \| t3D-12 \| \| t3D-13 \| \| t3D-14 \| \| t3D-15 \| \| t3D-16 \| \| t3D-17 \| \| t3D-18 \| \| t3D-19 \| \| t3D-20 \| \| t2M-1 \| \| t2M-2 \| \| t2M-3 \| \| t2M-4 \| \| t2M-5 \| \| t2M-6 \| \| t2M-7 \| \| t2M-8 \| \| t2M-9 \| \| t2M-10 \| \| t2M-11 \| \| t2M-12 \| \| t2M-13 \| \| t2M-14 \| \| t2M-15 \| \| t2M-16 \| \| t2M-17 \| \| t2M-18 \| \| t2M-19 \| \| t2M-20 \| \| t3M-1 \| \| t3M-2 \| \| t3M-3 \| \| t3M-4 \| \| t3M-5 \| \| t3M-6 \| \| t3M-7 \| \| t3M-8 \| \| t3M-9 \| \| t3M-10 \| \| t3M-11 \| \| t3M-13 \| \| t3M-14 \| \| t3M-15 \| \| t3M-16 \| \| t3M-17 \| \| t3M-19 \| \| t3M-20 \| \| t6M-1 \| \| t6M-2 \| \| t6M-3 \| \| t6M-4 \| \| t6M-5 \| \| t6M-6 \| \| t6M-7 \| \| t6M-8 \| \| t6M-9 \| \| t6M-10 \| \| t6M-11 \| \| t6M-13 \| \| t6M-14 \| \| t6M-15 \| \| t6M-16 \| \| t6M-17 \| \| t6M-18 \| \| t6M-19 \| \| t6M-20 \| \| t9M-1 \| \| t9m-3 \| \| t9m-4 \| \| t9m-5 \| \| t9m-6 \| \| t9m-8 \| \| t9m-10 \| \| t9m-11 \| \| t9m-13 \| \| t9m-15 \| \| t9m-16 \| \| t9m-18 \| \| t9m-19 \| \| t9m-20 \| | \| 158897 \| \| --- \| \| 34792 \| \| 47295 \| \| 36099 \| \| 49682 \| \| 114896 \| \| 107011 \| \| 101951 \| \| 111801 \| \| 110544 \| \| 101444 \| \| 27590 \| \| 33987 \| \| 62873 \| \| 100611 \| \| 75285 \| \| 135673 \| \| 117309 \| \| 110663 \| \| 64707 \| \| 156886 \| \| 121053 \| \| 103909 \| \| 51547 \| \| 58334 \| \| 37218 \| \| 12804 \| \| 29191 \| \| 80153 \| \| 79932 \| \| 24591 \| \| 132257 \| \| 111073 \| \| 60153 \| \| 33822 \| \| 104861 \| \| 140651 \| \| 54223 \| \| 20374 \| \| 159592 \| \| 95577 \| \| 119940 \| \| 17760 \| \| 121710 \| \| 133114 \| \| 122225 \| \| 93906 \| \| 157030 \| \| 101082 \| \| 95163 \| \| 34243 \| \| 155969 \| \| 149718 \| \| 118458 \| \| 37087 \| \| 93722 \| \| 84546 \| \| 51056 \| \| 55088 \| \| 105978 \| \| 84398 \| \| 32270 \| \| 35161 \| \| 137431 \| \| 97290 \| \| 83473 \| \| 84788 \| \| 164128 \| \| 143359 \| \| 97208 \| \| 106145 \| \| 118944 \| \| 127245 \| \| 52110 \| \| 142246 \| \| 25522 \| \| 88976 \| \| 130113 \| \| 86473 \| \| 148496 \| \| 74922 \| \| 98394 \| \| 81698 \| \| 47939 \| \| 39535 \| \| 123413 \| \| 120922 \| \| 103680 \| \| 108103 \| \| 134014 \| \| 88238 \| \| 181694 \| \| 117713 \| \| 163512 \| \| 151590 \| \| 220769 \| \| 162779 \| \| 170208 \| \| 144651 \| \| 99035 \| \| 110702 \| \| 91397 \| \| 78036 \| \| 139880 \| \| 75297 \| \| 105761 \| \| 21097 \| \| 168392 \| \| 162330 \| \| 140953 \| | \| RR \| \| --- \| \| RR \| \| RR \| \| RR \| \| RR \| \| RM \| \| RM \| \| RM \| \| RM \| \| RM \| \| MR \| \| MR \| \| MR \| \| MR \| \| MR \| \| MM \| \| MM \| \| MM \| \| MM \| \| MM \| \| RR \| \| RR \| \| RR \| \| RR \| \| RR \| \| RM \| \| RM \| \| RM \| \| RM \| \| MR \| \| MR \| \| MR \| \| MR \| \| MR \| \| MM \| \| MM \| \| MM \| \| MM \| \| MM \| \| RR \| \| RR \| \| RR \| \| RR \| \| RR \| \| RM \| \| RM \| \| RM \| \| RM \| \| RM \| \| MR \| \| MR \| \| MR \| \| MR \| \| MR \| \| MM \| \| MM \| \| MM \| \| MM \| \| MM \| \| RR \| \| RR \| \| RR \| \| RR \| \| RR \| \| RM \| \| RM \| \| RM \| \| RM \| \| RM \| \| MR \| \| MR \| \| MR \| \| MR \| \| MM \| \| MM \| \| MM \| \| MM \| \| RR \| \| RR \| \| RR \| \| RR \| \| RR \| \| RM \| \| RM \| \| RM \| \| RM \| \| RM \| \| MR \| \| MR \| \| MR \| \| MR \| \| MM \| \| MM \| \| MM \| \| MM \| \| MM \| \| RR \| \| RR \| \| RR \| \| RR \| \| RM \| \| RM \| \| RM \| \| MR \| \| MR \| \| MR \| \| MM \| \| MM \| \| MM \| \| MM \| | \| reef \| \| --- \| \| reef \| \| reef \| \| reef \| \| reef \| \| reef \| \| reef \| \| reef \| \| reef \| \| reef \| \| mangrove \| \| mangrove \| \| mangrove \| \| mangrove \| \| mangrove \| \| mangrove \| \| mangrove \| \| mangrove \| \| mangrove \| \| mangrove \| \| reef \| \| reef \| \| reef \| \| reef \| \| reef \| \| reef \| \| reef \| \| reef \| \| reef \| \| mangrove \| \| mangrove \| \| mangrove \| \| mangrove \| \| mangrove \| \| mangrove \| \| mangrove \| \| mangrove \| \| mangrove \| \| mangrove \| \| reef \| \| reef \| \| reef \| \| reef \| \| reef \| \| reef \| \| reef \| \| reef \| \| reef \| \| reef \| \| mangrove \| \| mangrove \| \| mangrove \| \| mangrove \| \| mangrove \| \| mangrove \| \| mangrove \| \| mangrove \| \| mangrove \| \| mangrove \| \| reef \| \| reef \| \| reef \| \| reef \| \| reef \| \| reef \| \| reef \| \| reef \| \| reef \| \| reef \| \| mangrove \| \| mangrove \| \| mangrove \| \| mangrove \| \| mangrove \| \| mangrove \| \| mangrove \| \| mangrove \| \| reef \| \| reef \| \| reef \| \| reef \| \| reef \| \| reef \| \| reef \| \| reef \| \| reef \| \| reef \| \| mangrove \| \| mangrove \| \| mangrove \| \| mangrove \| \| mangrove \| \| mangrove \| \| mangrove \| \| mangrove \| \| mangrove \| \| reef \| \| reef \| \| reef \| \| reef \| \| reef \| \| reef \| \| reef \| \| mangrove \| \| mangrove \| \| mangrove \| \| mangrove \| \| mangrove \| \| mangrove \| \| mangrove \| |

**Supplementary Table S5.** PERMANOVA analysis of Amplicon Sequence Variant (ASVs) richness (Chao1) and diversity (Shannon’s) present in *Pocillopora acuta* between mangrove (n=10) and reef (n=10) environments prior to transplantation (t_0_). df = degrees of freedom; SS = sum of squares; MS = mean sum of squares; Pseudo-F = F-value by permutation, *p* = *p*-value.

| **Chao1** | **Source** | **df** | **SS** | **MS** | **Pseudo-F** | **R^2^** | ***p* (perm)** |
| --- | --- | --- | --- | --- | --- | --- | --- |
|  | **Sites** | 1 | 9698.8 | 9698.8 | 13.127 | .416 | 0.001 |
|  | **Residuals** | 18 | 13573 |  |  |  |  |
| **Shannon’s** | **Source** | **df** | **SS** | **MS** | **Pseudo-F** | **R^2^** | ***p* (perm)** |
|  | **Sites** | 1 | 1378.2 | 1378.2 | 19.443 | .519 | 0.001 |
|  | **Residuals** | 18 | 1275.9 |  |  |  |  |

**Supplementary Figure S1.** Bacterial community composition of the coral *Pocillopora acuta* shown as relative abundance (%) across transplantation treatments; mangrove-mangrove, mangrove-reef, reef-mangrove and reef-reef, and between timepoints t_0_ (prior to transplantation) and t_9M_ (nine months after transplantation). Each proportion is > 1% relative abundance of bacterial families based on 16S rRNA sequencing (Illumina MiSeq) that matched to one phylogenetic group from the Silva v138 database. Top grey bars represent all other families < 1% relative abundance. Each bar represents an individual replicate.

**Supplementary Table S6.** PERMANOVA analysis of bacterial communities (beta diversity) present between mangrove (n=10) and reef (n=10) environments at t_0_. df = degrees of freedom; SS = sum of squares; MS = mean sum of squares; Pseudo-F = F-value by permutation, *p* = *p*-value.

| **Source** | **df** | **SS** | **MS** | **Pseudo-F** | **R^2^** | ***p* (perm)** |
| --- | --- | --- | --- | --- | --- | --- |
| **Site** | 1 | 9969.1 | 9969.3 | 2.6004 | .126 | 0.001 |
| **Residuals** | 18 | 69007 | 3833.7 |  |  |  |

**Supplementary Table S7.** MetagenomeSeq analysis of significantly differentially abundant bacterial families (*p* < 0.05, following FDR corrections) present in the microbiome of *Pocillopora acuta* between mangrove (n=10) and reef (n=10) environments. Family name is provided where possible.

| **Taxonomic ID** | ***p*-value _(corrected)_** |
| --- | --- |
| \| Sulfurovaceae \| \| --- \| \| Endozoicomonadaceae \| \| Milano_WF1B_44 \| \| Desulfocapsaceae \| \| B2M28 \| \| Thermoanaerobaculaceae \| \| Woeseiaceae \| \| Methyloligellaceae \| \| UC_bacteria \| \| Cyanobiaceae \| \| Sporichthyaceae \| \| UBA10353 \| \| Thiomicrospiraceae \| \| Bacillaceae \| \| Anaerolineaceae \| \| Marinococcaceae \| \| Carnobacteriaceae \| \| Lachnospiraceae \| \| Rubritaleaceae \| \| Microbulbiferaceae \| \| Chromatiaceae \| \| KI89A \| \| Acaryochloridaceae \| \| Microtrichaceae \| \| Legionellaceae \| \| Thiotrichaceae \| \| Sedimenticolaceae \| \| Rhizobiales \| \| Vicinamibacteria \| \| Saprospiraceae \| \| KD4_96 \| \| Crocinitomicaceae \| \| Flavobacteriaceae \| \| Ectothiorhodospiraceae \| \| HOC36 \| \| Pseudomonadaceae \| \| Cyclobacteriaceae \| \| OM190 \| \| Sandaracinaceae \| \| Leuconostocaceae \| \| Burkholderiaceae \| \| Oxyphotobacteria \| \| Geoalkalibacteraceae \| \| Bacteroidetes \| \| Corynebacteriaceae \| \| Kiloniellaceae \| \| Micrococcaceae \| \| Oligoflexales \| \| Sphingomonadaceae \| \| Enterobacteriaceae \| \| Thermaceae \| \| Caldilineaceae \| \| Halomonadaceae \| \| PS1 \| \| Prevotellaceae \| \| Rhizobiaceae \| \| Ilumatobacteraceae \| \| PeM15 \| \| Francisellaceae \| \| Moraxellaceae \| | \| 8.27E-13 \| \| --- \| \| 8.27E-13 \| \| 9.21E-12 \| \| 1.89E-11 \| \| 4.19E-11 \| \| 6.32E-11 \| \| 3.88E-10 \| \| 3.98E-10 \| \| 5.37E-10 \| \| 6.01E-08 \| \| 1.89E-06 \| \| 2.12E-06 \| \| 2.12E-06 \| \| 2.59E-06 \| \| 3.29E-06 \| \| 3.90E-06 \| \| 4.94E-06 \| \| 1.07E-05 \| \| 1.25E-05 \| \| 1.31E-05 \| \| 1.31E-05 \| \| 1.31E-05 \| \| 1.31E-05 \| \| 1.32E-05 \| \| 2.01E-05 \| \| 2.88E-05 \| \| 3.40E-05 \| \| 4.90E-05 \| \| 5.49E-05 \| \| 8.57E-05 \| \| 0.00013393 \| \| 0.00013585 \| \| 0.00014514 \| \| 0.00015943 \| \| 0.00015943 \| \| 0.00017888 \| \| 0.00017888 \| \| 0.00024914 \| \| 0.00027583 \| \| 0.00034664 \| \| 0.0015407 \| \| 0.0018492 \| \| 0.0019321 \| \| 0.0024897 \| \| 0.0030128 \| \| 0.0038452 \| \| 0.0038452 \| \| 0.0055182 \| \| 0.0067008 \| \| 0.0067008 \| \| 0.0070076 \| \| 0.0081229 \| \| 0.010025 \| \| 0.014583 \| \| 0.014583 \| \| 0.016388 \| \| 0.027788 \| \| 0.038251 \| \| 0.042801 \| \| 0.045132 \| |

**Supplementary Figure S2.** Bacterial diversity of ASVs associated with *Pocillopora acuta* between transplantation time points (t_0_ and t_9M_) and treatments; mangrove-mangrove (MM), mangrove-reef (MR), reef-mangrove (RM) and reef-reef (RR). Data based on chao1 and Shannon’s diversity index. Box plots represent 25^th^ to 75^th^ percentile range, lines show medians, error bars represent IQR and + represents the mean. N = 3-5 coral colonies (denoted with grey dots).

**Supplementary Table S8.** Two-factorial PERMANOVA analysis of Amplicon Sequence Variant (ASVs) richness (Chao1) and diversity (Shannon’s) present in *Pocillopora acuta* between transplantation time points (t_0_ and t_9M_) of treatments; mangrove-mangrove (MM), mangrove-reef (MR), reef-reef (RR) and reef-mangrove (RM). df = degrees of freedom; SS = sum of squares; MS = mean sum of squares; Pseudo-F = F-value by permutation, *p* = *p*-value.

| **Chao1 index** | | | | | | | | | |
| --- | --- | --- | --- | --- | --- | --- | --- | --- | --- |
| **Main PERMANOVA Test** | | | | | | | **Pairwise (t_0_-t_9M_)** | | |
| **Source** | **df** | **SS** | **MS** | **Pseudo F** | **R^2^** | ***p* (perm)** | **Groups** | ***t*** | ***p* (perm)** |
| **Site** | 3 | 7334.3 | 2444.8 | 3.6118 | .226 | 0.005 | MM | 1.1784 | 0.209 |
| **Time** | 1 | 720.19 | 720.19 | 1.064 | .022 | 0.335 | MR | 1.5035 | 0.172 |
| **Site x timepoints** | 3 | 6736.2 | 2245.4 | 3.3173 | .208 | 0.012 | RR | 2.1051 | 0.063 |
| **Residuals** | 26 | 17599 |  |  |  |  | RM | 1.7588 | 0.088 |
| **Shannon’s Index** | | | | | | | | | |
| **Main PERMANOVA Test** | | | | | | | **Pairwise (t_0_-t_9M_)** | | |
| **Source** | **df** | **SS** | **MS** | **Pseudo F** | **R^2^** | ***p* (perm)** | **Groups** | ***t*** | ***p* (perm)** |
| **Site** | 3 | 270.97 | 90.324 | 4.1134 | .224 | 0.011 | MM | 1.2355 | 0.268 |
| **Time** | 1 | 3.7964 | 3.7946 | 0.17281 | .003 | 0.715 | MR | 4.3222 | 0.021 |
| **Site x timepoints** | 3 | 363.17 | 121.06 | 5.5129 | .30 | 0.005 | RR | 1.1569 | 0.276 |
| **Residuals** | 26 | 570.92 |  |  |  |  | RM | 0.1688 | 0.127 |

**Supplementary Table S9.** Two-factorial PERMANOVA analysis of bacterial communities (beta diversity) associated with *Pocillopora acuta* between transplantation time points ( t_0_, t_3D_, t_2M_, t_3M_, t_6M_, t_9M_) of treatments; mangrove-mangrove (MM), mangrove-reef (MR), reef-reef (RR) and reef-mangrove (RM), and additional comparisons of treatments; (RM and MM) and (MR and RR) at timepoint t_9M_. df = degrees of freedom; SS = sum of squares; MS = mean sum of squares; Pseudo-F = F-value by permutation, *p* = *p*-value. All pairwise comparison *p*-values have undergone FDR corrections.

| **Main PERMANOVA test** | | | | | | | | | | |
| --- | --- | --- | --- | --- | --- | --- | --- | --- | --- | --- |
| **Source** | | **df** | **SS** | **MS** | | **Pseudo-F** | | **R^2^** | | ***p* (perm)** |
| **Sites** | | 1 | 9969.1 | 9969.3 | | 2.6004 | | .102 | | .001 |
| **Timepoints** | | 5 | 8867 | 8933.2 | | 2.4501 | | .091 | | .001 |
| **Sites x timepoints** | | 6 | 9925 | 9934.2 | | 2.5498 | | .102 | | .001 |
| **Residuals** | | 18 | 69007 |  | |  | |  | |  |
| **Pairwise** | | | | | | | | | | |
| **Factor** |  | | | | **Groups tested** | | ***t*** | | ***p* (perm) _corrected_** | |
| **Sites** | **Mangrove-mangrove** | | | |  | |  | |  | |
|  |  |  |  |  | t_0_-t_3D_ | | 1.3092 | | .037 | |
|  |  |  |  |  | t_0_-t_2M_ | | 1.2039 | | .010 | |
|  |  |  |  |  | t_0_-t_3M_ | | 1.2105 | | .018 | |
|  |  |  |  |  | t_0_-t_6M_ | | 1.2387 | | .008 | |
|  |  |  |  |  | t_0_-t_9M_ | | 1.2092 | | .014 | |
|  | **Mangrove-reef** | | | |  | |  | |  | |
|  |  |  |  |  | t_0_-t_3D_ | | 1.3609 | | .038 | |
|  |  |  |  |  | t_0_-t_2M_ | | 1.209 | | .021 | |
|  |  |  |  |  | t_0_-t_3M_ | | 1.1261 | | .026 | |
|  |  |  |  |  | t_0_-t_6M_ | | 1.1233 | | .055 | |
|  |  |  |  |  | t_0_-t_9M_ | | 1.1177 | | .021 | |
|  | **Reef-reef** | | | |  | |  | |  | |
|  |  |  |  |  | t_0_-t_3D_ | | 1.1816 | | .112 | |
|  |  |  |  |  | t_0_-t_2M_ | | 1.3436 | | .035 | |
|  |  |  |  |  | t_0_-t_3M_ | | 1.1455 | | .110 | |
|  |  |  |  |  | t_0_-t_6M_ | | 1.245 | | .041 | |
|  |  |  |  |  | t_0_-t_9M_ | | 1.1155 | | .133 | |
|  |  |  |  |  |  | |  | |  | |
|  | **Reef-mangrove** | | | | t_0_-t_3D_ | | 1.1052 | | .302 | |
|  |  |  |  |  | t_0_-t_2M_ | | 1.1119 | | .109 | |
|  |  |  |  |  | t_0_-t_3M_ | | 1.306 | | .046 | |
|  |  |  |  |  | t_0_-t_6M_ | | 1.2437 | | .037 | |
|  |  |  |  |  | t_0_-t_9M_ | | 1.2014 | | .032 | |
| **Timepoint** | **t_9M_** | | | |  | |  | |  | |
|  |  |  |  |  | RM-MM | |  | | .709 | |
|  |  |  |  |  | MR-RR | |  | | .401 | |

**Supplementary Table 10.** MetagenomeSeq analysis of significantly differentially abundant bacterial families (*p* < 0.05, following FDR corrections between timepoints (t_0_ vs t_9M_) in reef-mangrove *Pocillopora acuta* colonies. Family name is provided where possible.

| **Taxonomic ID** | ***p*-value _corrected_** |
| --- | --- |
| \| Endozoicomonadaceae \| \| --- \| \| Microcystaceae \| \| Pseudomonadaceae \| \| Francisellaceae \| \| Lachnospiraceae \| \| Thermaceae \| \| Rhizobiaceae \| \| Halomonadaceae \| \| Microtrichaceae \| \| Ectothiorhodospiraceae \| \| Micrococcaceae \| \| Amoebophilaceae \| \| Hyphomicrobiaceae \| \| Thiotrichaceae \| \| Bacillaceae \| \| Kiloniellaceae \| \| Rubritaleaceae \| | \| 0.00043821 \| \| --- \| \| 0.0012067 \| \| 0.0037592 \| \| 0.0082632 \| \| 0.0082632 \| \| 0.0082632 \| \| 0.020627 \| \| 0.023417 \| \| 0.024735 \| \| 0.024735 \| \| 0.024735 \| \| 0.024735 \| \| 0.024735 \| \| 0.025288 \| \| 0.027044 \| \| 0.042917 \| \| 0.048142 \| |

**Supplementary Table S11.**  MetagenomeSeq analysis of significantly differentially abundant bacterial families (*p* < 0.05, following FDR corrections between timepoints (t_0_ vs t_9M_) in mangrove-reef ) *Pocillopora acuta* colonies. Family name is provided where possible.

| **Taxonomic ID** | ***p*-value _corrected_** |
| --- | --- |
| \| Milano_WF1B_44 \| \| --- \| \| Endozoicomonadaceae \| \| Cyanobiaceae \| \| Halieaceae \| \| Anaerolineaceae \| \| Desulfocapsaceae \| \| Methyloligellaceae \| \| Marinococcaceae \| \| Kiloniellaceae \| | \| 0.0058214 \| \| --- \| \| 0.0058214 \| \| 0.0058214 \| \| 0.0058214 \| \| 0.012298 \| \| 0.015457 \| \| 0.015994 \| \| 0.030384 \| \| 0.045709 \| |
